# 非小细胞肺癌podoplanin阳性淋巴管密度与多层螺旋CT表现的关系

**DOI:** 10.3779/j.issn.1009-3419.2012.01.07

**Published:** 2012-01-20

**Authors:** 晖 周, 曾 熊, 进康 刘, 胜喜 陈, 漠玲 周, 腾宇 刘洋

**Affiliations:** 1 410008 长沙，中南大学湘雅医院放射科 Department of Radiology, Xiangya Hospital, Central South University, Changsha 410008, China; 2 410008 长沙，中南大学湘雅医院心胸外科 Department of Cardiothoracic Surgery, Xiangya Hospital, Central South University, Changsha 410008, China

**Keywords:** 肺肿瘤, 体层摄影术, X线计算机, 免疫组织化学, 微淋巴管密度, Lung neoplasms, Tomography, X-ray computed, Immunohistochemical technique, Lymphatic microvessel density

## Abstract

**背景与目的:**

现有的研究表明：肺癌微淋巴管密度（lymphatic microvessel density, LMVD）与淋巴结转移密切相关，但与肺癌多层螺旋CT（multi-slice spiral computed tomography, MSCT）的影像学改变的相关性尚不十分清楚。本研究通过podoplanin标记非小细胞肺癌患者手术标本微淋巴管并计数LMVD，观察患者肺癌病灶MSCT表现。

**方法:**

对34例非小细胞肺癌术前行MSCT检查，收集相关临床病理结果；评价MSCT表现（包括边缘形态、内部结构、邻近结构的CT征象）；免疫组织化学SP法检测肿瘤组织中心区、周边区的LMVD。

**结果:**

MSCT表现有棘状突起、胸膜凹陷征和癌性淋巴管炎的患者，其肺癌切除标本周围区LMVD均高于无上述表现者（*P*均 < 0.05）。

**结论:**

MSCT出现棘状突起、胸膜凹陷征或癌性淋巴管炎表现提示更高的肿瘤淋巴管生成水平，具有更高的淋巴结转移风险。

## Abstract (en)

**Background and objective:**

It has been proven that ymphatic microvessel density (LMVD) was closely correlated with the lymphatic metastasis of non-small cell lung cancer (NSCLC). The aim of the present study is to explore the relationship between podoplanin-LMVD and multi-slice spiral computed tomography (MSCT) characteristics of NSCLC.

**Methods:**

MSCT scanning was performed on 34 cases of NSCLC (squamous carcinoma, 15 cases; adenocarcinoma, 15 cases; and adenosquamous carcinoma, 4 cases) prior to operation. Clinical pathology results, including lymph node metastasis, were obtained. CT characteristics, such as shape of the edge, internal structure, and adjacent structures, were described. LMVD in the central and peripheral areas examined respectively using SP immunohistochemical technique were analyzed.

**Results:**

Lymph node metastasis was found to be associated with LMVD in the peripheral areas. LMVD in the peripheral areas of the resected lesions, the MSCT findings of which included spinous process, pleural indentation, and carcinomatous lymphangitis, was higher than that of the lesions without these MSCT characteristics (*P* < 0.05).

**Conclusion:**

MSCT findings of spinous process, pleural indentation, or carcinomatous lymphangitis of NSCLC may suggest a higher level of tumor lymphangiogenesis with a higher risk of lymph node metastasis.

影响可切除性原发性非小细胞肺癌（non-small cell lung cancer, NSCLC）患者生存最重要的因素是淋巴结的转移，目前有研究^[1]^表明肿瘤微淋巴管密度（lymphatic microvessel density, LMVD）与淋巴结转移有关，但对它与影像学的关系还了解不多，本研究用免疫组化的方法检测34例NSCLC患者手术标本内微淋巴管特异性标志物podoplanin的表达，分析其与NSCLC淋巴转移及肺癌结节多层螺旋CT（multi-slice spiral computed tomography, MSCT）表现的关系。

## 1 资料与方法

### 1.1 一般资料

选择2005年12月-2009年3月我院经MSCT扫描、肺癌切除术和病理证实的NSCLC病例34例，均为肺内单发结节，结节直径2.0 cm-3.0 cm；患者术前未行化疗或放疗等抗肿瘤治疗，在CT扫描后1周内手术；其中男性25例，女性9例；年龄44岁-73岁，平均58.3岁；按第7版AJCC肺癌TNM分期标准，p-TNM分期Ⅰ期12例，Ⅱ期9例，Ⅲ期13例。组织学分类：鳞癌15例，腺癌（含肺泡细胞癌）15例，腺鳞癌4例。病理证实无淋巴结转移15例，有淋巴结转移19例。高分化9例，中分化12例，低分化13例。

### 1.2 CT扫描和征象分析

采用Philips公司Brilliance型16层螺旋CT机。扫描范围自肾上腺水平至肺尖，平静呼吸下屏气时扫描。主要技术参数为：120 kVp，100 mAs-250 mAs，探测器组合16 mm×1.5 mm，螺距值0.6-0.9，重建层厚2 mm，重建间隔1 mm，重建矩阵512×512。原始图像数据传输到工作站（MXV Philips）后，以结节为固定中心进行多平面重建技术（multiplanar reconstruction, MPR）操作，利用多种窗技术显示结节内部及结节-肺界面情况。利用表面遮盖法（shaded surface display, SSD）、最大密度投影法（maximum intensity projection, MIP）、容积再现技术（volume rendering technique, VRT）等三维成像技术进一步观察病灶形态与周围结构关系，多种后处理技术的结合利用更全面、立体、准确的评价结节的CT表现特征^[2]^。由2位具有5年以上胸部CT阅片经验的放射科诊断医师以双盲法按统一标准评价病灶的MSCT征象，存在分歧时通过协商达成一致。本研究纳入的MSCT征象分析指标参照国内外文献公认的标准^[3]^，选取能有效反映结节的生物学特征并被广泛认同的以下指标：①边缘形态（毛刺、浅分叶、深分叶、棘状突起）；②结节内部结构（空泡征、实性结节、部分实性结节）；③结节邻近结构（胸膜凹陷征、血管聚集征、阻塞性表现、癌性淋巴管炎）。MSCT图像上测量肺门及各组纵隔淋巴结的短径，短径若＜1 cm则判为淋巴结肿大。

### 1.3 免疫组织化学染色及结果判断

将已取到的肿瘤组织经4%甲醛溶液固定，常规石蜡包埋，厚4 µm连续切片制备涂胶白片供HE染色和免疫组织化学染色备用。行常规HE染色，观察恶性结节病理形态学变化并根据2004年WHO肺癌组织学分类标准^[4]^进行分类和分级。使用试剂为鼠抗人podoplanin单克隆抗体（克隆号：H2907，美国Santa Cruz公司）、兔抗人血管内皮生长因子（vascular endothelial growth factor, VEGF）抗体（克隆号：SP28，北京中杉金桥生物技术有限公司）、鼠抗人增殖细胞核抗原（proliferation cell nucler antigen, PCNA）抗体（克隆号：PC10，北京中杉金桥生物技术有限公司）。实验方法严格按照说明书操作，采用链霉素抗生物素蛋白-过氧化氢连接（SP）二步法进行免疫组织化学染色显示肿瘤微淋巴管。以邻近的正常肺实质内的微淋巴管为阳性内对照，并参照试剂订购公司提供的阳性结果照片，PBS代替一抗为空白对照。肿瘤微淋巴管判断标准及微淋巴管密度计数方法参照Weidner等^[1, 5, 6]^报道的方法并加以改进，LMVD分中心区（结节最大层面的中心部分，测量点距肿瘤中心的距离占肿瘤半径的50%），周围区（中心区以外距结节边缘2 mm-3 mm以内的环状区域），计数时先在低倍镜（×100）下观察全层肿瘤组织，确定区域内微淋巴管密度最大的“热点”，每区随机抽取4个热点，保证每个计数区域采样均匀，每张切片共读取8个热点。于200 mm×0.74 mm视野下人工计数热点的微淋巴管数目，取其平均值为各区域的LMVD。邻近的正常肺实质内的微淋巴管不做计数对象，只作内对照评价免疫组织化学染色。所有标本的计数由2位经验丰富的病理科医师在不同的时间计数3次，选择2次相同或相近数目的平均数。VEGF、PCNA表达结果判断采用文献通用的方法^[7, 8]^。

### 1.4 统计学分析

以SPSS 19.0统计软件进行数据分析。计量资料统计结果均以Mean±SD表示；若各组方差齐同，样本均数比较采用两独立样本*t*检验或单因素方差分析；若方差不齐且无法校正，用*t*秩和检验；多个指标的相关分析采用*Spearmen*相关分析；以*P*＜0.05为差异有统计学意义。

## 2 结果

### 2.1 肺结节CT表现及三维重建技术

横断位原始图像结合三维重建技术能全面、细致地表现出肺结节形态特征，更直观地显示病灶与周围组织结构的空间立体关系（图 1A-图 1D）；毛刺征19例；浅分叶9例，深分叶25例；棘状突起10例；空泡征6例；实性结节25例，部分实性结节9例，未发现非实性结节；胸膜凹陷征28例；血管聚集征24例；阻塞改变（阻塞性肺炎或节段性不张）5例；癌性淋巴管炎16例；34例肺结节内均未见空洞及脂肪。

**1:**
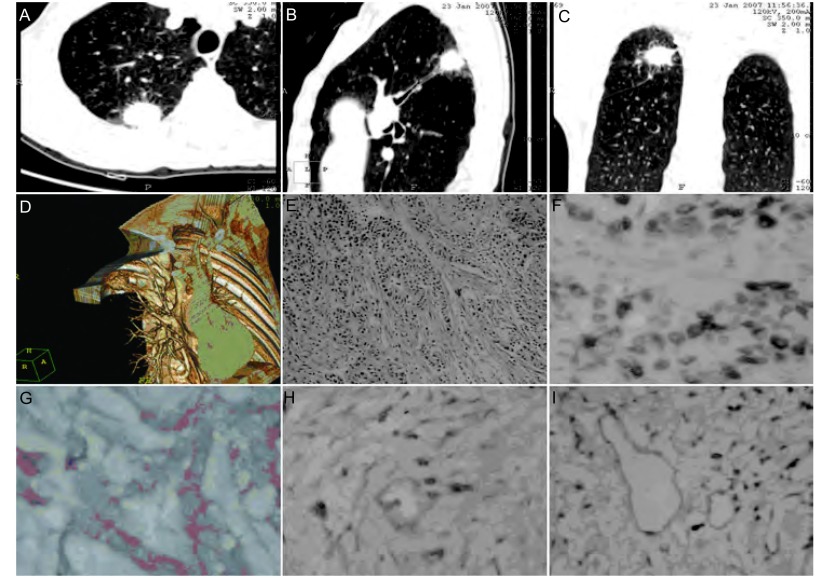
57岁男性右上肺NSCLC患者。MSCT横轴位（A）显示毛刺征，MPR技术显示分叶征和结节后下方阻塞病变（B）、胸膜凹陷征和棘状突起（C），VRT技术显示血管聚集征（D）。组织病理HE染色：低分化腺鳞癌（×200）；免疫组化PCNA、VEGF染色均呈强阳性表达（×200，F，G）；podoplanin染色见中心区微淋巴管稀少（×400，H），癌间质内少量podoplanin表达（×400，H），周围区微淋巴管较多，管腔扩大且不规则（×400，I）。 NSCLC found in the right upper lobe of a 57-year-old male. Axial images of MSCT shows spicules (A), MPR shows lobulated sign and distal side obstructive change (B), pleural indentation, and spinous process (C), VRT shows vessel convergence (D). HE staining of histopathology: poorly differentiated adenosquamous carcinoma (×200); Strongly positive expressions of PCNA, VEGF (SP×400, F, G); Lymphatic microvessels in the central areas were rare (podoplanin, SP×400, H), podoplanin were also expressed in few tumor stromata (SP×400, H), lymphatic microvessels in the peripheral areas were more with irregular and enlarged lumina (podoplanin, SP×400, I).

### 2.2 肺癌微淋巴管密度与CT表现

Podoplanin阳性表达的淋巴管多数位于NSCLC组织的边缘区，少数位于鳞癌巢周缘、腺癌的癌间质内。癌组织中心区淋巴管数目较少，大部分扩张不明显，可闭塞成条索状；癌周组织淋巴管密度增多，大部分不规则明显扩张且管壁结构不完整，围绕癌组织成环状分布（图 1D-I）。周围区LMVD（17.6±3.7）高于中心区LMVD（10.0±2.8）（*P*＜0.001）。存在病理性淋巴结转移组肺结节的周围区（20.2±3.5）明显大于无淋巴结转移组（15.5±2.0）（*P*=0.008），存在病理性淋巴结转移组肺结节的中心区LMVD（10.2±3.7）与无淋巴结转移组（9.8±1.8）无明显差异（*P*=0.062）；有淋巴结肿大和无淋巴结肿大两组之间各区域LMVD均无明显差异（*P*均＞0.05）。*Spearmen*相关分析显示淋巴结转移与肺门、纵隔淋巴结肿大无关（*P*＞0.05）；周围区LMVD与VEGF表达强度、PCNA阳性率均正相关，相关系数分别为0.600、0.570（*P*均＜0.01）；中心区LMVD与VEGF表达强度和PCNA阳性率均不相关（*P*均＞0.05）。

肺癌结节MSCT表现与肿瘤组织LMVD关系见表 1。有棘状突起征组、有胸膜凹陷征组、有癌性淋巴管炎组的周围区LMVD均高于无此征象组的周围区LMVD，组间差异有统计学意义；实性结节组和部分实性结节组之间及有毛刺征组、有分叶征组、有空泡征组、有血管聚集征组、有阻塞病变组与无此表现组周围区LMVD比较，组间差异无统计学意义；中心区LMVD在各表现不同组之间差异均无统计学意义。

**1:** 肺癌结节MSCT表现与肿瘤组织podoplanin阳性LMVD关系 The relationship between CT characteristics and podoplanin-LMVD

MSCT characteristics			*n*	LMVD in the central areas	*P*	LMVD in the peripheral areas	*P*
Edge of the shape	Spicules	(+)	19	9.1±2.0	0.353	15.5±2.8	0.079
		(-)	15	10.2±3.2		14.6±3.7	
	Lobulated sign	Shallow	25	10.1±2.8	0.635	17.3±4.6	0.148
		Deep	9	9.7±2.6		16.0±3.2	
	Spinous process	(+)	10	11.0±1.0	0.097	17.6±3.8	0.029
		(-)	24	9.8±2.8		14.5±2.3	
Internal structure	Vacuole	(+)	6	11.5±1.6	0.472	15.5±0.5	0.217
		(-)	28	9.7±2.9		16.0±3.9	
	Attenuation density	Solid nodules	9	10.0±2.9	0.071	17.6±3.5	2.319
		Part-solid nodules	25	10.0±2.6		17.3±4.3	
Adjacent structures	Pleural indentation	(+)	28	10.3±2.9	0.402	20.5±4.9	0.012
		(-)	6	8.5±1.6		16.9±3.1	
	Vessel convergence	(+)	24	10.6±0.8	0.316	16.4±3.0	0.087
		(-)	10	8.9±1.1		15.3±1.6	
	Obstructive change	(+)	5	10.1±2.9	0.081	17.6±3.8	0.065
		(-)	29	9.0±0.3		17.0±0.6	
	Carcinomatous lymphangitis	(+)	16	10.5±3.3	0.063	18.2±4.9	< 0.001
		(-)	18	9.4±2.0		14.9±0.8	
Lymph node	Enlargement	(+)	13	10.1±3.5	0.071	17.6±3.6	0.989
		(-)	21	9.8±0.4		17.5±3.9	
	Metastasis	(+)	15	10.2±3.7	0.062	20.2±3.5	0.008
		(-)	19	9.8±1.8		15.5±2.0	
MSCT: multi-slice spiral computed tomography; LMVD: lymphatic microvessel density.							

## 3 讨论

恶性肿瘤主要通过血道和淋巴道形成远处转移，是影响疗效和患者死亡的重要原因，但大部分恶性肿瘤最初发生的转移并不是通过血管，而是淋巴道^[9]^。目前对于肺癌结节CT表现与肿瘤生物学特征相关的研究多集中于血管生成方面，本研究使用已被证实具有良好特异性的淋巴管内皮细胞标志物podoplanin^[1, 10]^来检测肿瘤LMVD，探讨肺癌结节MSCT表现与肿瘤淋巴管生成之间的关系。

肿瘤淋巴管生成是指在肿瘤原位形成新的毛细淋巴管，由于肿瘤新生淋巴管结构特殊，使得肿瘤细胞更容易进入淋巴管道形成转移^[9, 10]^。我们的研究结果显示NSCLC组织中的微淋巴管主要存在于周边区域，中心区域淋巴管较少而且多呈闭塞状态，而周围区微淋巴管大部分不规则明显扩张且管壁结构不完整；分区域计数LMVD后与淋巴结转移关系分析结果显示与淋巴结转移有关的是周围区LMVD，中心区LMVD与淋巴结转移无关。Liang等^[11]^的研究也认为淋巴道转移主要是通过癌周区微淋巴管内皮细胞的开放和癌细胞对淋巴管管壁的破坏而进入淋巴管管腔的。相对于单纯的横断位图像，MSCT多种后处理技术的综合运用能更好的显示肺癌病灶内部结构、边缘形态及与周围结构的关系^[2]^，本研究显示MSCT图像提示周围区LMVD较高的表现包括有棘状突起、胸膜凹陷征、癌性淋巴管炎，而肺癌的MSCT征象均与中心区LMVD无关。以上结果提示与转移相关的功能性淋巴管主要分布在肿瘤组织周边，而出现棘状突起、胸膜凹陷征、癌性淋巴管炎的表现提示肿瘤淋巴管生成旺盛，具有更高的淋巴结转移风险。

我们前期研究发现微血管构筑表型的网状结构调控肿瘤血管新生与肿瘤增殖^[7, 12]^，本研究还发现周围区LMVD既和VEGF表达强度正相关又和PCNA阳性率正相关。目前普遍认为VEGF家族通过VEGF/VEGF-R2途径诱导血管形成，而通过VEGF-C/VEGF-D/VEGF-R3途径诱导淋巴管形成^[13, 14]^。近年来有研究^[15, 16]^表明VEGF除了明显促进血管新生之外还有较强的促淋巴管生成和促淋巴结转移作用，VEGF和VEGF-C均是强大的内皮调控因子，可以诱导肿瘤内微血管和微淋巴管内皮细胞增殖，两者之间可能存在协同作用^[13, 14, 17]^。VEGF是微血管和淋巴管通透性增高的主要因素，VEGF表达越高，微血管和淋巴管内皮细胞连接越松散^[15]^，因此越容易发生癌细胞的转移。PCNA的表达是评价细胞增殖能力的主要指标，这些细胞同时包括肿瘤细胞、血管内皮细胞和淋巴管内皮细胞，从而可以评价肿瘤细胞诱导血管和淋巴管新生的能力。本实验结果表明微血管构筑表型的网状结构不仅调控血管新生与肿瘤增殖，同时也调控淋巴管生成。

我们的研究还分析了淋巴结肿大与转移的关系，结果显示两者无关，而且有淋巴结肿大和无淋巴结肿大两组之间各区域LMVD均无明显差异，合并肺炎或患有增殖性病变均可导致淋巴结肿大，而没有肿大的淋巴结可能已有转移。因此，术前仅靠淋巴结形态学的检查来判断是否存在转移有一定的误差，提示我们还需要结合肺癌淋巴管生成状态来对淋巴结转移的风险进行综合评价。本研究发现棘状突起、胸膜凹陷、癌性淋巴管炎等MSCT表现与肿瘤淋巴管生成有一定的相关性，出现这些征象的肺癌可能生长活跃、恶性程度高、较容易出现淋巴结转移，该结果可为无创性评估肿瘤淋巴管生成状态提供有意义的参考。
